# A predictive algorithm for identifying children with sickle cell anemia among children admitted to hospital with severe anemia in Africa

**DOI:** 10.1002/ajh.26492

**Published:** 2022-02-16

**Authors:** Peter Olupot-Olupot, Roisin Connon, Sarah Kiguli, Robert O. Opoka, Florence Alaroker, Sophie Uyoga, Margret Nakuya, William Okiror, Julius Nteziyaremye, Tonny Ssenyondo, Eva Nabawanuka, Juliana Kayaga, Cynthia Williams Mukisa, Denis Amorut, Rita Muhindo, Gary Frost, Kevin Walsh, Alexander W. Macharia, Diana M. Gibb, A. Sarah Walker, Elizabeth C. George, Kathryn Maitland, Thomas N. Williams

**Affiliations:** 1 Busitema University Faculty of Health Sciences, Mbale Regional Referral Hospital, Mbale, Uganda; 2 Mbale Clinical Research Institute, Mbale, Uganda; 3 Medical Research Council Clinical Trials Unit (MRC CTU), University College London, London, UK; 4 Department of Paediatrics and Child Health, School of Medicine, Makerere University, Kampala, Uganda; 5 Soroti Regional Referral Hospital, Soroti, Uganda; 6 Kenya Medical Research Institute (KEMRI)-Wellcome Trust Research Programme, Kilifi, Kenya; 7 Section for Nutrition Research, Department of Metabolism, Digestion and Reproduction, Imperial College, London, UK; 8 Department of Surgery and Cancer, Institute of Global Health and Innovation, Imperial College, London, UK

## Abstract

Sickle cell anemia (SCA) is common in sub-Saharan Africa where approximately 1% of births are affected. Severe anemia is a common cause for hospital admission within the region yet few studies have investigated the contribution made by SCA. The Transfusion and Treatment of severe anemia in African Children Trial (ISRCTN84086586) investigated various treatment strategies in 3983 children admitted with severe anemia (hemoglobin < 6.0 g/dl) based on two severity strata to four hospitals in Africa (three Uganda and one Malawi). Children with known-SCA were excluded from the uncomplicated stratum and capped at 25% in the complicated stratum. All participants were genotyped for SCA at trial completion. SCA was rare in Malawi (six patients overall), so here we focus on the participants recruited in Uganda. We present baseline characteristics by SCA status and propose an algorithm for identifying children with unknown-SCA. Overall, 430 (12%) and 608 (17%) of the 3483 Ugandan participants had known- or unknown-SCA, respectively. Children with SCA were less likely to be malaria-positive and more likely to have an affected sibling, have gross splenomegaly, or to have received a previous blood transfusion. Most outcomes, including mortality and readmission, were better in children with either known or unknown-SCA than non-SCA children. A simple algorithm based on seven admission criteria detected 73% of all children with unknown-SCA with a number needed to test to identify one new SCA case of only two. Our proposed algorithm offers an efficient and cost-effective approach to identifying children with unknown-SCA among all children admitted with severe anemia to African hospitals where screening is not widely available.

## 1 Introduction

Severe anemia is among the leading causes of hospital admission and death among children in many parts of sub-Saharan Africa.^1^ Historically, the contribution of sickle cell anemia (SCA) to pediatric admissions for severe anemia in Africa has been underappreciated, probably reflecting the lack of diagnostic services.^2^ However, its importance has been highlighted in more recent studies, including two that were conducted in Kilifi county in Kenya which found that the incidence of hospital admission with severe anemia (hemoglobin [Hb] < 5.0 g/dl) was between 30 and 60 times higher in children with than without SCA, and that children with SCA receive between 10% and 20% of all transfusions administered to children.^3,4^ These statistics are particularly striking given the relatively low prevalence of SCA at birth within the Kilifi region (0.8%).^4^

The Transfusion and Treatment of severe anemia in African Children Trial (TRACT; ISRCTN84086586) investigated a range of management strategies among children admitted to hospitals in Africa with severe anemia.^5,6^ A substantial proportion of those recruited were affected by SCA, either already known or diagnosed subsequently by genotyping conducted at the end of the trial. Here, we describe the enrolment characteristics and subsequent outcomes of TRACT participants stratified by SCA group. We also describe a diagnostic algorithm aimed at identifying children with unknown-SCA from among all children admitted to hospitals in Africa with severe anemia.

## 2 Methods

### 2.1 The TRACT

TRACT was an open-label, multicentre, factorial, randomized controlled trial recruiting children aged 2 months to 12 years presenting with severe anemia (Hb < 6.0 g/dl) to four hospitals in Africa, three in Uganda (Mulago, Mbale, and Soroti) and one in Malawi (in Blantyre). Full details of the trial justification and design^7^ and the results of previous analyses^5,6,8–10^ have been described in detail previously. Briefly, in the complicated stratum, children with an Hb < 6.0 g/dl and one or more severity features (which included known-SCA) were randomized 1:1 to immediate transfusion with 30 versus 20 ml/kg whole blood, or the equivalent volumes as packed cells. To increase generalizability to regions of lower SCA prevalence, recruitment of children with known-SCA was capped at 25%. In the uncomplicated stratum, children with an Hb of 4.0–6.0 g/dl and no severity features were randomized 1:1:2 to 30 versus 20 ml/kg whole blood (or the equivalent in packed cells) versus to no immediate transfusion (control). Children with known SCA were not recruited to the uncomplicated stratum, which included a “no immediate transfusion” arm (although transfusion could be triggered by de novo severity signs or Hb < 4.0 g/dl), because the investigators were not in equipoise regarding the withholding of transfusions from children with known-SCA and Hb levels of 4.0–6.0 g/dl. Children in both strata were further randomized to receive either 3 months of postdischarge antibacterial chemoprophylaxis with cotrimoxazole, or not, and 3 months of nutritional support with either iron and folate (standard of care) or with multivitamin–multimineral mix.^11^

### 2.2 Laboratory methods

Full blood counts (by use of automated analysers) and malaria tests (by either microscopy of Giemsa-stained blood films or rapid diagnostic tests; RDTs) were conducted on all participants at recruitment. An extra sample of blood was collected from children with suspected bacterial sepsis and inoculated into culture bottles (BACTEC Peds Plus; Becton Dickinson) as previously described.^12^ Culture bottles were weighed before and after blood inoculation and processed by an automated blood-culture system (BACTEC 9050; Becton Dickinson). Positive samples were subcultured on standard media by routine microbiological techniques. Quality assurance was provided by the UK National External Quality Assessment Service. Clinical indications for lumbar puncture were impaired consciousness or meningism in children younger than 5 years, prostration in children younger than 3 years, seizures (other than febrile seizures) in children younger than 2 years, and suspicion of sepsis in children younger than 60 days. All admitted children were also tested for HIV by use of RDTs. Children were then reviewed at 28-, 90- and 180-day postrecruitment, when Hb levels were remeasured by either full blood counts (Beckman Coulter) or by calibrated point-of-care tests (HemoCue Hb301 system; HemoCue AB).^13^ At trial completion, all participants were genotyped for the rs334 β^s^ polymorphism in HBB (the cause of SCA) by polymerase chain reaction using genomic DNA extracted from EDTA blood samples collected at recruitment (and therefore pre-transfusion) and stored at −80°C, a method described in detail previously.^14^ SCA genotypes were defined as follows: ancestral-type homozygotes at rs334 (AA); heterozygotes (sickle cell trait; AS;) and rs334-derived homozygotes (SCA; SS). In Uganda, other major forms of sickle cell disease caused by the coinheritance of the β^s^ mutation and either HbC or β-thalassaemia have not been reported while other forms are very rare.^15^ As a result, our focus was on HbSS.

### 2.3 Statistical analysis

Children were divided into four SCA groups for analysis: AA, AS, with SS further categorized into (a) those whose diagnosis was already known at admission (including six children who were diagnosed during their admission) (known-SCA) and (b) those whose diagnosis was only revealed by the retrospective batch genotyping of admission blood samples after completion of the trial (unknown-SCA). Baseline characteristics were summarized by these groups and compared using rank sum or exact tests for continuous and categorical variables respectively. The effect of SCA on a range of outcomes was then investigated: 28- and 180- day mortality, readmissions for all cause and three specific causes (anemia, malaria, and DUS), time to discharge, change in Hb at 8 h and 180 days, and receiving two or more blood transfusions. Analysis of each outcome adjusted for potential confounders. This analysis used published prognostic models for 28- and 180-day mortality in TRACT^5^ and readmissions.^10^ Otherwise, new models were built using backwards elimination (exit *p* value .1) on all children enrolled in TRACT to maximize power to identify confounders. Potential covariates included demographic details, vital signs on admission, clinical history of the illness, results of laboratory tests at admission, information on the trial arm and trial transfusions, and time and date of admission. Continuous variables were modeled as fractional polynomials using the mfp command in Stata with α = .05. Time from discharge to readmission for any cause, and for malaria and anemia specifically, was modeled treating death as a competing risk. Time to discharge was modeled treating death in hospital as a competing risk. Change in Hb at 8 h and 180 days was modeled using linear regression, adjusting for baseline Hb. We used logistic regression to model the probability of having two or more transfusions among those children who were transfused at least once. For all outcomes, the final model was refitted to Ugandan children only, and the four-level variable for SCA group was added to the model if not already selected.

### 2.4 Predicting unknown-SCA among TRACT participants

In addition, we built a multivariable logistic regression model to identify predictors of unknown-SCA among Ugandan trial participants. Children with known-SCA were excluded from this analysis. Our model included clinical factors, malaria status, full blood count data and levels of the inflammatory marker C-reactive protein (CRP). Variable selection was performed using backwards elimination with exit *p* = .1. We then simplified the model by removing factors that were only weakly predictive or that are not routinely collected in Africa outside of research to develop a risk score aimed at identifying children with unknown-SCA among all children admitted with severe anemia. We categorized continuous variables based on known clinical cut-offs or the shape of the association with the outcome. Each factor was assigned a score proportional to its coefficient in the model. From this risk score, we calculated the receiver operating characteristic (ROC) along with the sensitivity, specificity, positive predictive value (PPV), negative predictive value (NPV) using various thresholds. Finally, we also calculated the number needed to test to diagnose one new case of SCA (NNT) among all children admitted with severe anemia.

### 2.5 Ethics

Either written informed consent or verbal assent with delayed written informed consent^16^ was obtained before randomization within the trial, which was stratified by centre and severity stratum. The trial protocol was approved by the Imperial College London Research Ethics Committee (ICREC_13-1-11) in the United Kingdom, by the Uganda National Committee for Science and Technology (UNCST), the Makerere University School of Medicine Research and Ethics Committee (#REC ref 2013–050) and the National Drug Authority in Uganda and by the College of Medicine Research and Ethics Committee in Malawi (P.03/13/1365).

## 3 Results

From September 17, 2014 to May 15, 2017, 3983 eligible children were randomly assigned to treatment, and followed up for 180 days. The sample flow is summarized in Figure S1. Results for SCA genotyping were available for 3944 (99%). Children without results for SCA genotyping were excluded from further analyses. In the complicated stratum 463/3157 (15%) parents reported SCA at admission and 886 (28%) were found to have SCA by genotype while 340/1549 (22%) children in the uncomplicated stratum were found to have SCA by retrospective genotyping. Only 6 out of the 461 (3.5%) children recruited to the study in Malawi overall had either known- or unknown-SCA overall, so we focussed our analysis on the 3483 children recruited in Uganda, where the frequency was considerably higher. This consisted of 1502 participants in Mbale, 1065 in Soroti, and 916 in Mulago. Among these children, 2321 (67%) had HbAA, 124 (4%) had HbAS, 430 (12%) had known-SCA, and 608 (17%) had unknown-SCA.

### 3.1 Admission characteristics

The admission characteristics of Ugandan trial participants, stratified by SCA category, are shown in Tables 1 and S1. The groups varied considerably in age, the median being 63 months in those with known-SCA compared to only 19 months in those with unknown-SCA and 35 and 31 months in AA and AS children respectively. Overall, 64% of children tested positive for malaria. Of these, 47% tested positive on both RDT and microscopy, 52% were positive on RDT only, and 1% were positive by microscopy only. Those with either known- or unknown-SCA were more likely than AA children to have received a previous blood transfusion (*p* < .001), to have gross splenomegaly (*p* < .001 and *p* = .006, respectively), or to have an affected sibling (*p < .001).* Conversely, they were less likely to test positive for malaria or to manifest neurological signs, had lower CRPs and had a poorer nutritional status. They were also less likely to be HIV positive, although the HIV prevalence was low at only 67 (2%) overall. We found no difference in the prevalence of invasive bacterial infections between the groups overall (Table S2) although in all cases the numbers involved were too small to lead to substantial conclusions.

### 3.2 The impact of SCA on mortality and other outcomes

Most outcomes were no worse while some were more favorable in SCA than AA children. Mortality by Day 28 was significantly lower in children with unknown-SCA (hazard ratio: 0.41; 0.19-0.88; *p* = .02) and in both SCA groups by Day 180 (*p* < .001 for both comparisons) (Table 2). The risk of readmission was similar among unknown-SCA and AA children for all-cause, anemia-specific and malaria-specific diagnoses (*p* > .45). The risk of readmission for all-causes or with severe anemia was significantly lower in those with known-SCA (Table 2). In contrast, a trend was seen towards increased mortality in AS children, which reached significance by Day 180 (Table 2). There was no evidence of association between SCA status and time to hospital discharge (Table 2). The odds of having a second transfusion were similar between the known-SCA and AA groups but were significantly lower in the unknown-SCA group (Table 2).

**Figure 1:**
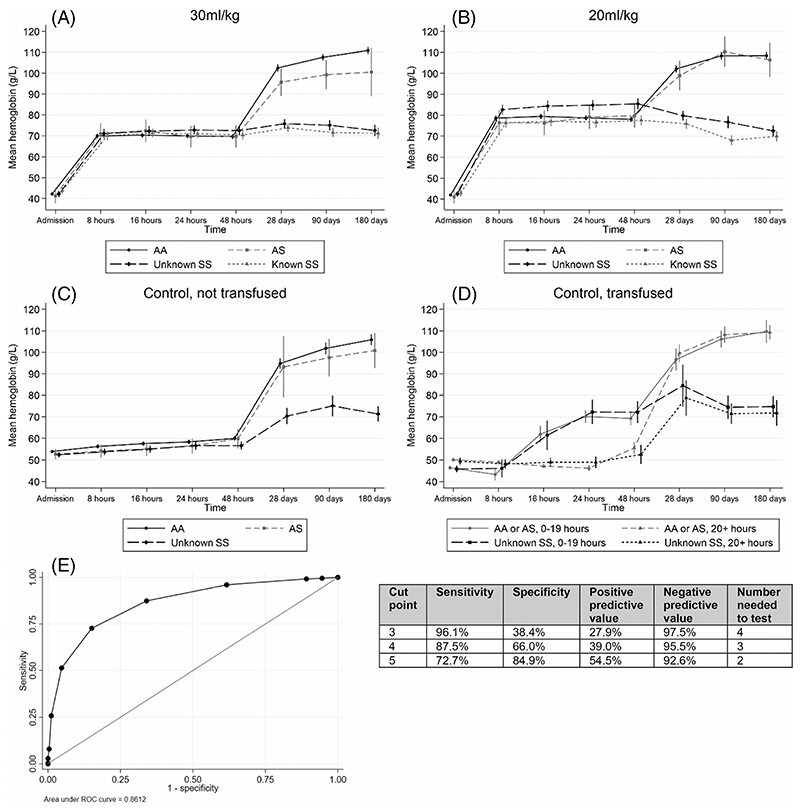
Hemoglobin recovery by trial arm and SCA status. (A and B) Mean hemoglobin (g/L) over time in by SCA group and transfusion randomization. Data for the control arm is shown separately for children who went on to have a triggered transfusion (C and D). Total N = 1380 in 30 ml/kg, N = 1393 in 20 ml/kg, N = 347 in control, not transfused, N = 363 in control, transfused. Children with known SCA at enrolment were not eligible for the control arm. (D) Results separately according to the time from randomization until children were transfused. The AS and AA groups were combined due to small numbers of AS patients. (E) Receiver operating characteristic (ROC) curve for model to predict unknown-SCA. ROC curve and summary statistics were considered positive for SCA if scored above the “Cut point”, with the true status defined according to genotype results (SS = positive, AA/AS = negative). A score of 5 was selected as the optimal cut point based on sensitivity and PPV. The number needed to test was calculated as 1/PPV. PPV, positive predictive value; SCA, sickle cell anemia

While the change in Hb between baseline and 8 h was similar across the SCA groups, in comparison to AA children, recovery was 3.6 and 3.9 g/dl lower by Day 180 among children with unknown- and known-SCA, respectively (*p* < .001 for both comparisons) (Table 2). All groups had a similar change in Hb during the first 48 h in both trial strata, at which point recovery stalled within the SCA groups, leaving both significantly more anemic at Day 28 (Figure 1A-D). Ninety-two percent of children with SCA had an Hb of < 10.0 g/dl at Day 28, compared to only 40% of those with AS or AA (odds ratio = 19.3; 13.9-26.9). Multivariable models for all outcomes are provided in Tables S3-S7 and a summary of the outcomes is given in Table S8.

### 3.3 Predicting unknown-SCA

Table 3 describes the factors predicting unknown-SCA status among TRACT participants. Compared to those without SCA (AA and AScombined), unknown-SCA was significantly more common in children <12 months of age and those with a negative malaria test. SCA was also associated with a high white blood cell count, a higher mean corpuscular volume, lower platelet counts, and with a history of SCA within the family. The maximum possible risk score, calculated from the total of the model coefficients, was 10. The median score in those with unknown-SCA was 6 (interquartile range [IQR]: 4–7), compared to 3 (IQR: 2–4) in those without SCA. The area under the ROC curve was 0.86 (Figure 1E). A risk score of ≥5 was associated with a sensitivity of 73%, specificity of 85%, PPV of 55%, and NPV of 93% in predicting children with unknown-SCA (Figure 1E). Using a score above this value equated to a NNT to identify one new SCA case of 2. Results from the full multivariable model for unknown-SCA are shown in Table S9.

## 4 Discussion

As the largest transfusion trial yet reported from Africa, TRACT offers a valuable opportunity to investigate the contribution of SCA to childhood hospital admissions for severe anemia within the region. Even though children with known-SCA were excluded from the uncomplicated stratum of the trial, and their numbers capped at 25% in the complicated arm, almost one third of all participants from Uganda had SCA overall. Among those whose SCA status was unknown at recruitment, 20% were subsequently found to have SCA in posttrial testing, providing an opportunity to consider potential approaches to identifying such children from within this population. Given that the birth prevalence of SCA within the study areas varies between only 0.7% and 1.5%,^17,18^ our findings also demonstrate that SCA contributes disproportionately to the burden of severe acute childhood anemia at these Ugandan study sites. The low proportion of HbAS children among study recruits (only 4% compared to the national prevalence of 13.3%^18^) presumably reflects the extraordinary protection afforded by this condition against *Plasmodium falciparum* malaria.

Even at steady state, children with SCA are severely anemic, with Hb concentrations that typically range between 6.0 and 8.0 g/dl. Sudden Hb drops, so-called anemic crises, are a common complication,^19^ the causes of which include episodes of red cell hyper-hemolysis or sequestration, both of which are most often precipitated by infections, and self-limiting aplastic crises caused by Parvovirus B19 infection.^19^ The role of malaria in the pathophysiology of anemic crises is controversial. While it is likely that, as in sickle cell trait, SCA reduces the frequency and severity of malaria infections, it also seems likely that malaria can precipitate episodes of sequestration or hemolysis to result in sudden and catastrophic declines in Hb.^20,21^ In TRACT, the prevalence of malaria was significantly lower in children with either AS or SCA than it was in AA children, suggesting that other causes of anemia such as bacterial or viral infections or nutritional causes may have played a more important role in these two patient groups. Nevertheless, given that malaria can still trigger anemic crises, it is important that children with known-SCA should be protected from malaria using chemoprophylaxis and other preventive measures.^21^

Marked age differences were seen between the groups, children with unknown-SCA being significantly younger and those with known-SCA being considerably older than AA children. It is likely that the former is explained by the natural history of anemic crises, which first start to present after approximately 3 months of age, the result of falling levels of hemoglobin F,^19^ while the latter is almost certainly attributable by delays in diagnosis. No national programme of neonatal or early life screening for SCA was in place in Uganda when the trial was conducted, so that most of those with known-SCA will have been tested on the basis of clinical suspicion, often following recurrent illness events. Once diagnosed, many will then have been under regular clinical follow-up where they will have received education about their disease and the need for prophylaxis and early treatment for malaria and bacterial infections. Together, these issues make it difficult to compare the baseline features and outcome between this and other groups. Despite this, the relatively poor nutritional status of both SCA groups in comparison to AA children is noteworthy. The causes of malnutrition among children with SCA are multifactorial and probably involve increased energy requirements due to a raised basal metabolic rate, altered metabolic pathways, the increased degradation and loss of nutrients, and reduced dietary intake, potentially from the anorexic effects of comorbidities.^22^ As such, malnutrition is a major, but potentially preventable, cause of poor outcomes among children living with SCA in sub-Saharan Africa^23^ and a priority area for future research.^24^

Of note, most outcomes were no worse among children in either of the SCA groups than they were in AA children, while some, including overall mortality, were more favorable. While this could suggest that standard emergency treatments for severe anemia are equally beneficial in both SCA and AA children, it might also indicate specific underlying health problems in the latter group. Similarly, we were surprised to find that postdischarge mortality was higher in AS than in AA children. Although this may be due to chance, given the relatively small number of children within that subgroup, it could equally result from the differing etiologies of severe anemia. Although malaria was probably the cause of the anemia in most AA children, only half of AS children tested positive for malaria at recruitment. Other more serious and less responsive under-lying conditions may, therefore, have played a disproportionate role in the AS children. Given the well-recognized risk of invasive bacterial infections in children with SCA^25^ the fact that we saw no difference in prevalence between the groups is perhaps surprising. Nevertheless, the low proportion of positive cultures overall probably reflects the limited specialized blood culture facilities that were available at the participating sites at the time the study was conducted. Moreover, vaccines against two of the most common pathogens that affect children with SCA, *Streptococcus pneumoniae* and *Haemophilus influenzae*,^25^ had already been rolled out within the study region across all under 5 year olds, suggesting that these vaccines may already be specifically helping to improve the survival of children with this condition.

A striking observation from our current analysis is the marked difference in the profiles of hematological recovery between children in the SCA and non-SCA groups. The relative rise in Hb levels was indistinguishable between these groups during the initial phase of active treatment and transfusion management; however, while it continued to rise in those without SCA it then stalled in the SCA groups as they reached their baseline values. This offers one potential opportunity for directing targeted testing towards children with unknown-SCA: a full blood count taken 1 month after discharge following hospital treatment for severe anemia could be highly informative, a diagnosis of SCA being highly likely in children with an Hb of <10.0 g/dl.

While newborn screening would clearly be the best approach and considerable progress in this regard has been made in Uganda during recent years. A pilot national screening programme, organized through the Uganda Central Public Health Laboratories was established in 2014, which was initially focused on the repurposing of dried blood spot samples collected to screen for the mother-to-child transmission of HIV.^18,26,27^ More recently, new approaches to early detection are starting to become available, including a number of promising rapid diagnostic methods.^28^ While such approaches show great promise, for the time being there is still a need for alternative approaches to opportunistic testing. In this study, we found that 20% of children admitted to hospitals in Uganda with severe anemia had unknown-SCA. If all such children were targeted for screening the NNT for each case detected would therefore be 5. We explored alternative, potentially more efficient approaches, to targeting such opportunistic testing by developing a diagnostic algorithm based on data that is commonly collected at admission. We derived a score that could potentially allow > 70% of those with unknown-SCA to be detected with a NNT of < 2. This would be a substantially more efficient approach to targeted testing in terms of costs and time and offers a potential compromise in areas where extensive testing may not be feasible. Given the recent development of accurate and low-cost point of care diagnostic methods for rapid SCA testing,^28^ there has never been a better time to step up opportunistic screening in hospitals.

In summary, a high proportion of all children with severe anemia who were recruited to the TRACT trial in Uganda had either known- or unknown-SCA. Standard case management was equally effective in these children as it was in those without SCA.^29^ We present an algorithm that could allow the detection of more than 70% of all children with undiagnosed SCA among those admitted with severe anemia with a NNT of less than 2. Implementing our algorithm could present a cost-efficient approach to the targeted testing of such children that could help direct those found to be affected to appropriate care.

## Supplementary Material

Supplemental File

## Figures and Tables

**Table 1:** Baseline characteristics at recruitment to the trial

Factor	AA (*N* = 2321, col % or median [IQR])	AS (*N* = 124, col % or median [IQR])	AS vs. AA *p* value	Unknown-SCA (*N* = 608, col % or median [IQR])	Unknown-SCA vs. AA *p* value	Known-SCA (*N* = 430, col % or median [IQR])	Known-SCA vs. AA *p* value
Demographics							
Age (months)	35 (18, 59)	31 (18, 52)	.53	19 (10, 51)	<.001	63 (30, 95)	<.001
Sex (% Males)	1314 (57%)	73 (59%)	.63	319 (52%)	.07	236 (55%)	.52
MUAC for age z-score (*n* = 3453)	−0.9 (−1.6, −0.3)	−1.0 (−1.6, −0.4)	.47	−1.2 (−2.0, −0.5)	<.001	−1.8 (−2.6, −0.9)	<.001
Transfused (ever) before this illness (*n* = 3473)	663 (29%)	40 (33%)	.36	209 (34%)	.006	318 (74%)	<.001
Number of siblings (*n* = 3476)	3 (1, 5)	3 (1, 5)	.59	2 (1, 4)	<.001	3 (2, 5)	.06
Sibling with SCA (*n* = 3134)	34 (2%)	5 (4%)	.046	60 (11%)	<.001	93 (24%)	<.001
Site							
Mbale	1053 (45%)	56 (45%)	.23	216 (36%)	<.001	177 (41%)	.008
Mulago	554 (24%)	37 (30%)		237 (39%)		88 (20%)	
Soroti	714 (31%)	31 (25%)		155 (25%)		165 (38%)	
Vital signs							
Respiratory distress	448 (19%)	25 (20%)	.82	111 (18%)	.60	71 (17%)	.18
Impaired consciousness	426 (18%)	18 (15%)	.34	74 (12%)	<.001	31 (7%)	<.001
Heart rate (beats/minute)	148 (133, 160)	144 (129, 157)	.08	148 (130, 164)	.64	133 (118, 148)	<.001
Temperature (°C)	37.2 (36.7, 37.8)	37.2 (36.8, 37.8)	.42	37.5 (36.8, 38.1)	<.001	37.4 (36.8, 38.1)	<.001
Respiratory rate (per minute)	40 (33, 49)	40 (32, 48)	.40	42 (35, 52)	<.001	36 (30, 46)	<.001
Clinical history of presenting illness							
Vomiting (*n* = 3478)	1501 (65%)	77 (62%)	.56	291 (48%)	<.001	171 (40%)	<.001
Fits in this illness (*n* = 3475)	181 (8%)	7 (6%)	.49	24 (4%)	<.001	4 (1%)	<.001
Splenomegaly (*n* = 3478)							
Not palpable	1485 (64%)	85 (69%)	.61	369 (61%)	.006	261 (61%)	<.001
Enlarged	694 (30%)	32 (26%)		180 (30%)		111 (26%)	
Gross	138 (6%)	7 (6%)		59 (10%)		57 (13%)	
Admitted into another hospital for >24 h (*n* = 3480)	365 (16%)	24 (19%)	0.31	64 (11%)	<.001	51 (12%)	.04
Laboratory tests							
Hemoglobin (g/dl)	4.5 (3.6, 5.4)	4.6 (3.6, 5.3)	.94	4.5 (3.8, 5.3)	.78	4.5 (3.6, 5.2)	.30
White blood cell count (×10^9^/L) (*n* = 3410)	12 (8, 20)	15 (9, 24)	.005	28 (19,39)	<.001	27 (19, 42)	<.001
MCV (fl) (*n* = 3429)	78 (73, 85)	74 (67, 81)	<.001	80 (74, 89)	<.001	87 (79, 95)	<.001
Platelets (10^9^/L) (*n* = 3425)	160 (88, 284)	232 (115, 366)	.002	202 (126, 307)	<.001	229 (134, 368)	<.001
Malaria positive (by blood slide or rapid diagnostic test) (*n* = 3480)	1815 (78%)	63 (51%)	<.001	180 (30%)	<.001	167 (39%)	<.001
Glucose (mmol/L) (*n* = 3447)	5.6 (4.9, 6.3)	5.5 (4.8, 6.4)	.80	5.6 (5.0, 6.3)	.17	5.6 (5.1, 6.3)	.10
Lactate (mmol/L) (*n* = 3458)	2.7 (1.9, 4.2)	2.7 (1.8, 4.0)	.33	2.5 (1.8, 3.5)	<.001	2.2 (1.6, 3.5)	<.001
HIV positive (*n* = 3308)	56 (3%)	5 (4%)	.22	5 (1%)	.01	1 (0%)	.001
CRP (mg/L) (*n* = 3321)	68 (28, 123)	45 (15, 77)	<.001	35 (12, 74)	<.001	39 (17, 75)	<.001
Positive blood culture^a^ (*n* = 3058)	71/2027 (4%)	3/117 (3%)	.80	23/540 (4%)	.44	9/374 (2%)	.35

Note: Total number included is 3483 (Uganda only); where a factor contains some missing data, the total nonmissing is shown in parentheses in the first column. Continuous variables are summarized by median (IQR) and *p* values from rank sum tests. Categorical variables are summarized by frequency and proportion of SCA group with *p* values from exact tests. 95% of the children were recruited within 24 h of admission.

Abbreviations: CRP, C-reactive protein; IQR, interquartile range; MCV, mean corpuscular volume; MUAC, mid upper arm circumference; SCA, sickle cell anemia.

a The specific organisms involved are summarized in Table S2.

**Table 2:** The effect of SCA status on clinical outcomes

Outcome	Outcome measure	HR or coefficient (AS vs. AA) [95% CI]	*p* Value (AS vs. AA)	HR or coefficient (unknown-SCA vs. AA) [95% CI]	*p* Value (unknown-SCA vs. AA)	HR or coefficient (known-SCA vs. AA) [95% CI]	*p* Value (known- SCA vs. AA)
Mortality–28 days	Hazard ratio	1.88 (0.81, 4.35)	.14	0.41 (0.19, 0.88)	.02	0.59 (0.27, 1.27)	.17
Mortality - 180 days	Hazard ratio	1.95 (1.17, 3.24)	.01	0.42 (0.27, 0.67)	<.001	0.24 (0.14, 0.42)	<.001
Readmission–All cause	Subhazard ratio	1.12 (0.72, 1.76)	.60	0.92 (0.73, 1.16)	.48	0.62 (0.46, 0.83)	.001
Readmission–Malaria	Subhazard ratio	0.86 (0.35, 2.12)	.75	0.85 (0.48, 1.50)	.567	0.58 (0.29, 1.14)	.11
Readmission–Anemia	Subhazard ratio	1.20 (0.71, 2.02)	.50	0.90 (0.68, 1.19)	.45	0.45 (0.30, 0.66)	<.001
Time to discharge	Subhazard ratio	0.87 (0.71, 1.07)	.18	1.06 (0.97, 1.15)	.21	1.04 (0.94, 1.15)	.42
Change in Hb–8 h	Hemoglobin higher by (g/dl) (linear regression)	0 (−0.2, 0.2)	.95	0.1 (−0.1, 0.2)	.28	0 (−0.2, 0.1)	.52
Change in Hb–180 days	Hemoglobin (g/dl) higher by (linear regression)	−0.4 (−0.9, 0.1)	.10	−3.6 (−3.8, −3.3)	<.001	−3.9 (−4.2, −3.7)	<.001
Two or more transfusions	Odds ratio	0.60 (0.29, 1.24)	.17	0.43 (0.29, 0.63)	<.001	0.99 (0.70, 1.41)	.97

*Note:* Effect sizes from models adjusted for covariates shown in Tables S3-S7.

Abbreviations: CI, confidence interval; Hb, hemoglobin; HR, hazard ratio.

**Table 3:** Multivariable model for predictors of unknown-SCA and risk score

Predictor	Odds ratio (95% CI)	*p* Value	Score
Age			
<12 months	1.47 (1.10, 1.97)	.009	1
≥12 months	1.00 (1.00, 1.00)		0
Sibling with sickle cell disease	4.79 (2.72, 8.42)	<.001	1
Malaria negative (by rapid diagnostic test or blood slide)	7.97 (6.16, 10.32)	<.001	2
WBC			
<10 × 10^9^/L	1.00 (1.00, 1.00)	<.001	0
10−19 × 10^9^/L	6.19 (3.81, 10.06)		1
20−29 × 10^9^/L	16.95 (10.30, 27.92)		2
≥30 × 10^9^/L	37.31 (22.60, 61.58)		3
MCV			
<90 fl	1.00 (1.00, 1.00)	<.001	0
≥ 90 fl	2.58 (1.88, 3.53)		1
Platelets			
<50 × 10^9^/L	1.00 (1.00, 1.00)	<.001	0
50−250 × 10^9^/L	7.24 (3.52, 14.91)		2
≥250 × 10^9^/L	2.92 (1.42, 6.02)		1

Note: Odds ratios from logistic regression on binary outcome of unknown SCA vs. not SCA. Known SCA were excluded from this analysis. *N* = 2681. Abbreviations: CI, confidence interval; MCV, mean corpuscular volume; SCA, sickle cell anemia; WBC, white blood cells.

## Data Availability

The data that support the findings of this study are available from the corresponding author upon reasonable request.
