# Supplementary material for: A predictive algorithm for identifying children with sickle cell anemia among children admitted to hospital with severe anemia in Africa
Source: Am J Hematol. 2022 Feb 16;97(5):527–36. doi: 10.1002/ajh.26492 (PMC7612591; doi:10.1002/ajh.26492)
Supplement: Supplementary file 1 — Appendix S1:Supporting Information [file AJH-97-527-s001.docx]

**Supplementary Figure 1.** TRACT recruitment profile**.**

3,983 children recruited to TRACT in Uganda and Malawi

rs334 genotyping failed in 39

3,944 children successfully genotyped at rs334

461 children recruited in Malawi dropped because of a low rs334 A>T allele frequency

3,483 successfully genotyped children recruited in Uganda contribute to the analysis

HbAA

2,321 (67%)

HbAS

124 (4%)

Known-SCA

430 (12%)

Unknown-SCA

608 (17%)

**Supplementary Table 1.** Pathogens detected in blood cultures.

|  | **AA**  **(N=71)** | **AS**  **(N=3)** | **Unknown-SS**  **(N=23)** | **Known-SS**  **(N=9)** |
| --- | --- | --- | --- | --- |
| *E. coli* | 7 (10%) | 0 (0%) | 0 (0%) | 0 (0%) |
| *Enterobacter spp* | 4 (6%) | 1 (33%) | 0 (0%) | 1 (11%) |
| *Enterococcus spp* | 2 (3%) | 0 (0%) | 1 (4%) | 0 (0%) |
| *H. influenzae* | 1 (1%) | 0 (0%) | 0 (0%) | 0 (0%) |
| *Klebsiella spp* | 7 (10%) | 0 (0%) | 0 (0%) | 0 (0%) |
| *Proteus mirabilis* | 1 (1%) | 0 (0%) | 0 (0%) | 0 (0%) |
| *Pseudomonas aeruginosa* | 0 (0%) | 0 (0%) | 2 (9%) | 0 (0%) |
| *S. aureus* | 20 (28%) | 0 (0%) | 7 (30%) | 1 (11%) |
| *S. pneumoniae* | 2 (3%) | 1 (33%) | 2 (9%) | 2 (22%) |
| *Salmonella spp* | 24 (34%) | 1 (33%) | 9 (39%) | 3 (33%) |
| Other | 3 (4%) | 0 (0%) | 2 (9%) | 2 (22%) |

Percentages are out of number with positive blood culture.

**Supplementary Table 2.** Additional baseline characteristics at the point of screening.

| **Candidate predictor** | **AA (N=2,321, col % or median (IQR))** | **AS (N=124, col % or median (IQR))** | **AS vs AA p-value** | **Unknown-SCA (N=608, col % or median (IQR))** | **Unknown-SCA vs AA p-value** | **Known-SCA (N=430, col % or median (IQR))** | **Known-SCA vs AA p-value** |
| --- | --- | --- | --- | --- | --- | --- | --- |
| Demographics | | | | | | | |
| Height for age z-score (n=3,428) | -1.0 (-2.0, -0.1) | -1.0 (-1.8, -0.2) | 0.98 | -1.0 (-1.9, -0.1) | 0.76 | -1.3 (-2.2, -0.3) | 0.002 |
| Weight for age z-score (n=3,393) | -1.2 (-2.0, -0.5) | -1.3 (-2.1, -0.6) | 0.32 | -1.4 (-2.1, -0.7) | 0.010 | -1.5 (-2.2, -0.7) | <0.001 |
| Blood group (n=3,130) - |  |  |  |  |  |  |  |
| A  B  AB  O | 560 (27%)  539 (26%)  117 (6%)  854 (41%) | 26 (25%)  31 (30%)  5 (5%)  41 (40%) | 0.85 | 134 (25%)  97 (18%)  26 (5%)  270 (51%) | <0.001 | 115 (27%)  97 (23%)  17 (4%)  201 (47%) | 0.11 |
| Two or more hospital admission in past year prior to primary admission (n=3,468) | 518 (22%) | 32 (26%) | 0.38 | 116 (19%) | 0.10 | 226 (53%) | <0.001 |
| Type of homestead (n=3,479) -  Urban  Semi urban  Rural | 168 (7%)  228 (10%)  1924 (83%) | 10 (8%)  18 (15%)  96 (77%) | 0.20 | 97 (16%)  99 (16%)  410 (68%) | <0.001 | 49 (11%)  54 (13%)  326 (76%) | 0.002 |
| Vital signs | | | | | | | |
| Systolic blood pressure (n=3,481) | 91 (84, 98) | 91 (85, 98) | 0.96 | 94 (86, 100) | <0.001 | 98 (89, 104) | <0.001 |
| Diastolic blood pressure (n=3,479) | 53 (47, 61) | 54 (49, 62) | 0.30 | 56 (49, 63) | <0.001 | 58 (50, 66) | <0.001 |
| Oxygen saturation (n=3,479) | 98 (95, 99) | 98 (96, 99) | 0.97 | 97 (95, 98) | 0.14 | 97 (94, 98) | <0.001 |
| Capillary refill time >2 seconds | 515 (22%) | 27 (22%) | 1.00 | 145 (24%) | 0.38 | 115 (27%) | 0.045 |
| Clinical history of presenting illness | | | | | | | |
| History of cough (n=3,481) | 1495 (64%) | 70 (56%) | 0.08 | 385 (63%) | 0.60 | 275 (64%) | 0.87 |
| Increased work of breathing (n=3,467) | 474 (21%) | 24 (19%) | 0.82 | 140 (23%) | 0.18 | 93 (22%) | 0.56 |
| Indrawing on admission (n=3,481) | 306 (13%) | 15 (12%) | 0.89 | 88 (14%) | 0.42 | 59 (14%) | 0.76 |
| Diarrhea on admission (n=3,479) | 312 (13%) | 16 (13%) | 1.00 | 103 (17%) | 0.03 | 24 (6%) | <0.001 |
| Deep breathing (n=3,478) | 308 (13%) | 16 (13%) | 1.00 | 59 (10%) | 0.02 | 43 (10%) | 0.07 |
| Crackles (n=3,469) | 140 (6%) | 11 (9%) | 0.18 | 61 (10%) | <0.001 | 39 (9%) | 0.03 |
| Sunken eyes (n=3,482) | 151 (7%) | 12 (10%) | 0.19 | 25 (4%) | 0.03 | 19 (4%) | 0.10 |
| Cold hands (n=3,481) | 88 (4%) | 5 (4%) | 0.81 | 12 (2%) | 0.03 | 6 (1%) | 0.01 |
| Liver >2cm below costal margin (n=3,472) | 517 (22%) | 25 (20%) | 0.66 | 120 (20%) | 0.20 | 138 (32%) | <0.001 |
| Jaundice (n=3,480) | 822 (35%) | 45 (36%) | 0.85 | 183 (30%) | 0.01 | 252 (59%) | <0.001 |
| Received antihelminths in last 6 months (n=3,417) | 557 (25%) | 26 (21%) | 0.45 | 135 (22%) | 0.28 | 127 (30%) | 0.03 |
| Laboratory tests | | | | | | | |
| MCH (picogram) (n=3,430) | 25 (23, 28) | 24 (20, 27) | <0.001 | 26 (23, 28) | 0.032 | 27 (25, 30) | <0.001 |
| MCHC (g/dL) (n=3,423) | 32 (30, 34) | 32 (30, 34) | 0.65 | 31 (30, 33) | <0.001 | 31 (30, 33) | 0.002 |
| Lymphocytes (10^9^/L) (n=3,101) | 5 (3, 8) | 6 (3, 10) | 0.016 | 12 (7, 19) | <0.001 | 11 (7, 19) | <0.001 |
| Granulocytes (10^9^/L) (n=3,073) | 6 (3, 9) | 6 (3, 12) | 0.063 | 10 (6, 16) | <0.001 | 10 (6, 16) | <0.001 |
| Monocytes (10^9^/L) (n=3,095) | 0.9 (0.5, 1.5) | 1.1 (0.5, 1.8) | 0.17 | 1.9 (1.2, 3.1) | <0.001 | 1.9 (1.1, 3.1) | <0.001 |

**Supplementary Table 3.** Multivariable models for mortality.

| **Predictor** | **28-day mortality** | | **180-day mortality** | |
| --- | --- | --- | --- | --- |
|  | **Hazard ratio** | **p value** | **Hazard ratio** | **p value** |
| HIV positive | 1.80 (0.68, 4.79) | 0.24 | 1.39 (0.70, 2.79) | 0.35 |
| Blood group- A  B  AB  O | 1.00  0.66 (0.33, 1.30)  2.42 (1.16, 5.05)  1.08 (0.63, 1.85) | 0.015 | 1.00  0.80 (0.54, 1.18)  1.71 (1.03, 2.84)  1.03 (0.74, 1.43) | 0.039 |
| Oxygen saturation | 0.91 (0.87, 0.94) | <0.001 | 0.94 (0.91, 0.97) | <0.001 |
| Respiratory rate | 1.03 (1.01, 1.05) | 0.002 | 1.02 (1.01, 1.03) | 0.003 |
| Fits in this illness | 1.57 (0.83, 2.97) | 0.17 | 0.78 (0.47, 1.30) | 0.34 |
| Blantyre Coma Score - 5  4  3  2  1  0 | 1.00  1.17 (0.47, 2.95)  3.02 (1.28, 7.13)  2.67 (1.22, 5.85)  3.42 (0.84, 13.92)  3.59 (1.12, 11.51) | 0.021 | 1.00  1.22 (0.65, 2.29)  2.87 (1.60, 5.15)  2.20 (1.22, 3.97)  4.91 (1.56, 15.40)  2.38 (0.79, 7.12) | <0.001 |
| Lactate at admission (mmol/L) | 1.11 (1.04, 1.18) | 0.002 | 1.06 (1.02, 1.11) | 0.008 |
| Malaria (rapid diagnostic test or blood slide) | 0.50 (0.32, 0.78) | 0.003 | 0.42 (0.31, 0.56) | <0.001 |
| Site - Mbale  Mulago  Soroti |  |  | 1.00  1.04 (0.73, 1.47)  0.96 (0.69, 1.33) | 0.919 |
| Sickle genotype - AA  AS  SS |  |  | 1.00  1.96 (1.18, 3.25)  0.33 (0.23, 0.49) | <0.001 |
| Received blood transfusion ever, prior to this illness |  |  | 1.65 (1.24, 2.19) | <0.001 |

Hazard ratios from Cox regression models. Estimates were also adjusted for the transfusion volume (30ml/kg or 20ml/kg), temperature and the interaction between the two, modelled with natural cubic splines. Children in the control arm were excluded from these models. N=2,604 and 2,598 for 28-day and 180-day mortality respectively.

**Supplementary Table 4.** Multivariable models for readmissions.

| **Predictor** | **All cause readmissions** | **Anemia readmissions** | | | **Malaria readmissions** | |
| --- | --- | --- | --- | --- | --- | --- |
|  | **Subhazard ratio** | **p value** | **Subhazard ratio** | **p value** | **Subhazard ratio** | **p value** |
| Site - Mbale  Mulago  Soroti | 1.00  0.81 (0.65, 1.02)  0.99 (0.80, 1.21) | 0.16 | 1.00  0.55 (0.41, 0.74)  0.70 (0.54, 0.90) | <0.001 | 1.00  1.05 (0.63, 1.75)  1.03 (0.70, 1.52) | 0.97 |
| Age at primary admission (per year younger) | 1.07 (1.11, 1.03) | <0.001 | 1.04 (1.09, 1.00) | 0.048 | 1.18 (1.26, 1.10) | <0.001 |
| HIV positive | 2.73 (1.67, 4.46) | <0.001 | 3.16 (1.84, 5.44) | <0.001 | 0.48 (0.06, 3.70) | 0.48 |
| Sickle status - AA  AS  SS, unknown at discharge  SS, known at discharge | 1.00  1.12 (0.72, 1.76)  0.92 (0.73, 1.16)  0.62 (0.46, 0.83) | 0.012 | 1.00  1.20 (0.71, 2.02)  0.90 (0.68, 1.19)  0.45 (0.30, 0.66) | <0.001 | 1.00  0.86 (0.35, 2.12)  0.85 (0.48, 1.50)  0.58 (0.29, 1.14) | 0.45 |
| Two or more hospital admissions in the last year before primary admission | 1.44 (1.18, 1.75) | <0.001 | 1.70 (1.34, 2.16) | <0.001 | 1.26 (0.89, 1.79) | 0.19 |
| Received blood transfusion ever, prior to this illness | 1.40 (1.06, 1.86) | 0.019 | 1.54 (1.09, 2.16) | 0.013 | 1.92 (1.00, 3.70) | 0.049 |
| History of cough at primary admission | 1.12 (0.93, 1.34) | 0.23 | 1.04 (0.83, 1.31) | 0.71 | 1.09 (0.79, 1.50) | 0.59 |
| Indrawing on admission | 1.41 (1.11, 1.79) | 0.004 | 1.13 (0.84, 1.51) | 0.43 | 1.31 (0.85, 2.04) | 0.22 |
| Splenomegaly on admission - Not palpable  Enlarged  Gross | 1.00  1.22 (1.02, 1.47)  1.42 (1.05, 1.91) | 0.023 | 1.00  1.43 (1.14, 1.80)  1.66 (1.17, 2.37) | 0.001 | 1.00  1.18 (0.86, 1.63)  1.07 (0.59, 1.95) | 0.60 |
| Diarrhea on admission | 0.72 (0.54, 0.96) | 0.023 | 0.73 (0.51, 1.03) | 0.076 | 0.74 (0.43, 1.28) | 0.28 |
| Received oral antimalarials in last week before primary admission | 1.03 (0.87, 1.22) | 0.72 | 1.19 (0.97, 1.46) | 0.097 | 0.91 (0.68, 1.23) | 0.56 |
| Malaria positive at primary admission, no previous blood transfusion | 0.57 (0.44, 0.74) | <0.001 | 0.51 (0.36, 0.71) | <0.001 | 1.40 (0.77, 2.55) | 0.27 |
| Malaria positive at primary admission, previous blood transfusion | 1.96 (1.39, 2.75) | <0.001 | 1.44 (1.03, 2.01) | 0.004 | 2.75 (1.53, 4.96) | 0.95 |
| Randomized >24h after admission | 1.37 (0.96, 1.95) | 0.08 | 1.48 (0.96, 2.27) | 0.076 | 0.94 (0.47, 1.88) | 0.87 |
| Strata - TRACT A  TRACT B, immediate transfusion  TRACT B, deferred transfusion  TRACT B, no transfusion | 1.00  0.92 (0.73, 1.17)  1.04 (0.78, 1.40)  0.70 (0.48, 1.01) | 0.25 | 1.00  0.90 (0.65, 1.25)  1.15 (0.79, 1.67)  0.68 (0.41, 1.15) | 0.31 | 1.00  1.07 (0.70, 1.64)  1.62 (0.98, 2.66)  0.59 (0.29, 1.20) | 0.044 |
| Age of blood pack (per week older) | 1.06 (0.98, 1.14) | 0.16 | 1.07 (0.98, 1.17) | 0.13 | 1.08 (0.95, 1.23) | 0.26 |
| Length of stay (per day longer) | 1.03 (1.00, 1.06) | 0.05 | 1.02 (0.98, 1.06) | 0.38 | 1.01 (0.96, 1.07) | 0.62 |
| Missed dose of MVMM, iron folate or cotrimoxazole by 28 days | 1.44 (1.21, 1.71) | <0.001 | 1.36 (1.10, 1.68) | 0.004 | 1.22 (0.89, 1.67) | 0.22 |
| Admitted >24 hours into another hospital at primary admission |  |  | 0.74 (0.54, 1.01) | 0.06 |  |  |
| Hemoglobin at admission (per g/L lower) |  |  | 4.01 (12.09, 1.33) | 0.014 |  |  |
| Type of homestead - Urban  Semi urban  Rural |  |  |  |  | 1.00  0.65 (0.18, 2.28)  2.72 (0.99, 7.43) | 0.004 |
| Able to walk unaided before this illness at primary admission |  |  |  |  | 1.87 (1.04, 3.39) | 0.038 |
| Temperature gradient at primary admission |  |  |  |  | 2.22 (1.33, 3.70) | 0.002 |
| Cotrimoxazole randomization- cotrimoxazole (vs no cotrimoxazole) |  |  |  |  | 0.79 (0.59, 1.06) | 0.11 |
| Monocytes (per 10^9^/L) at admission |  |  |  |  | 0.83 (0.73, 0.95) | 0.008 |
| Platelets (per 100 x 10^9^/L) at admission |  |  |  |  | 1.11 (1.01, 1.21) | 0.022 |
| Blood pack type- settled (vs whole) |  |  |  |  | 0.66 (0.46, 0.94) | 0.022 |

Subhazard ratios from competing risks models for time from discharge to first readmission, with death treated as a competing risk. Cause-specific models ignore readmissions for other causes. Total included N=3,150 for all-cause readmissions, N=3,147 for anemia readmissions and N=2,756 for malaria readmissions.

**Supplementary Table 5.** Multivariable model for time to discharge.

| **Predictor** | **Subhazard ratio (95% CI)** | **p value** |
| --- | --- | --- |
| Site - Mbale  Mulago  Soroti | 1.00  0.78 (0.70, 0.87)  0.55 (0.50, 0.60) | <0.001 |
| MUAC for age z-score (per unit higher) | 1.06 (1.03, 1.09) | <0.001 |
| HIV positive | 0.87 (0.69, 1.09) | 0.22 |
| Type of homestead- Urban  Semi urban  Rural | 1.00  0.93 (0.81, 1.08)  0.88 (0.77, 1.01) | 0.15 |
| Impaired consciousness | 0.73 (0.66, 0.82) | <0.001 |
| Oxygen saturation | 1.03 (1.02, 1.05) | <0.001 |
| History of fever for more than 14 days | 0.69 (0.59, 0.81) | <0.001 |
| Indrawing on admission | 0.85 (0.76, 0.95) | 0.004 |
| Fits in this illness | 0.81 (0.69, 0.94) | 0.007 |
| Deep breathing | 0.83 (0.74, 0.94) | 0.002 |
| Crackles | 0.83 (0.73, 0.95) | 0.009 |
| Sunken eyes | 0.90 (0.78, 1.04) | 0.16 |
| Cold hands | 0.76 (0.61, 0.94) | 0.012 |
| Liver >2cm below costal margin | 0.91 (0.84, 0.98) | 0.018 |
| Diarrhea on admission | 0.86 (0.78, 0.94) | 0.002 |
| Jaundice | 0.94 (0.88, 1.01) | 0.085 |
| Admitted for over 24 hours into another hospital | 0.87 (0.79, 0.96) | 0.004 |
| Received traditional medicine in last week | 0.88 (0.80, 0.97) | 0.008 |
| Hemoglobin at screening (per g/L lower) | 0.60 (0.82, 0.44) | 0.001 |
| Malaria (rapid diagnostic test or blood slide) | 1.09 (1.02, 1.16) | 0.011 |
| Positive blood culture at screening | 0.88 (0.75, 1.03) | 0.11 |
| Received antibiotics on admission | 0.89 (0.82, 0.96) | 0.003 |
| Had transfusion | 0.87 (1.00, 0.76) | 0.053 |
| Blood pack age (if transfused) (days) | 0.99 (0.99, 1.00) | 0.006 |
| Blood pack type- settled (vs whole) (if transfused) | 0.90 (0.83, 0.97) | 0.008 |
| Randomization- Control  20mls/kg  30mls/kg | 1.00  1.39 (1.26, 1.54)  1.58 (1.42, 1.75) | <0.001 |
| Time admitted- before 12:00  12:00-17:59  After 18:00 | 1.00  0.92 (0.86, 0.99)  0.84 (0.76, 0.92) | <0.001 |

Subhazard ratios for time from randomization to discharge with death before discharge as competing risk. N=2,804. 44 children died before discharge, 22 censored due to absconding before discharge.

**Supplementary Table 6.** Multivariable model for change in hemoglobin.

| **Predictor** | **8 hours** | | **180 days** | |
| --- | --- | --- | --- | --- |
|  | **Coefficient** | **p value** | **Coefficient** | **p value** |
| Site- Mbale  Mulago  Soroti | 0.00  0.27 (-1.33, 1.88)  2.73 (1.68, 3.78) | <0.001 | 0.00  0.84 (-1.57, 3.26)  6.92 (4.91, 8.92) | <0.001 |
| Age at admission (per year) |  |  | 0.87 (0.51, 1.23) | <0.001 |
| Age at admission (log) | -5.83 (-6.54, -5.13) | <0.001 |  |  |
| Sex (male vs female) | -0.95 (-1.75, -0.14) | 0.02 |  |  |
| Heart rate (log) | -2.08 (-2.82, -1.34) | <0.001 |  |  |
| Received blood transfusion ever, prior to this illness | -0.93 (-1.86, -0.01) | 0.047 | -2.98 (-5.02, -0.95) | 0.004 |
| Splenomegaly- Not palpable  Enlarged  Gross | 0.00  -0.96 (-1.88, -0.05)  -1.64 (-3.25, -0.03) | 0.03 |  |  |
| Hemoglobin at screening (per g/L) | 0.79 (0.75, 0.83) | <0.001 | 0.13 (0.05, 0.21) | 0.002 |
| Malaria (rapid diagnostic test or blood slide) | -1.10 (-1.95, -0.24) | 0.01 |  |  |
| Blood pack age (if transfused) (days) | -0.11 (-0.17, -0.06) | <0.001 |  |  |
| Blood pack hemoglobin (g/L) (square root) | 0.52 (0.45, 0.58) | <0.001 |  |  |
| Blood pack type- Whole blood | 0.00 (0.00, 0.00) | 0.002 |  |  |
| Packed cells (spun) | -3.20 (-6.43, 0.02) | 0.002 |  |  |
| Red Cell Concentrate (gravity) | -4.11 (-6.74, -1.48) | 0.002 |  |  |
| Transfusion volume - Control  20mls/kg  30mls/kg | 0.00  33.55 (32.05, 35.05)  43.81 (42.32, 45.31) | <0.001 |  |  |
| Interaction -  20mls/kg and packed cells (spun)  20mls/kg and Red Cell Concentrate (gravity)  30mls/kg and packed cells (spun)  30mls/kg and Red Cell Concentrate (gravity) | -9.99 (-13.42, -6.56)  -10.62 (-13.60, -7.64)  -9.88 (-13.33, -6.42)  -7.01 (-9.99, -4.02) | <0.001 |  |  |
| Able to walk without support before this illness |  |  | 2.46 (-0.07, 4.99) | 0.056 |
| Had transfusion |  |  | 3.68 (0.82, 6.54) | 0.01 |
| Sickle genotype- AA  AS  SS |  |  | 0.00 | <0.001 |
|  |  |  | -4.08 (-8.90, 0.75) |  |
|  |  |  | -37.15 (-39.01, -35.29) |  |
| Type of homestead- Urban  Semi urban  Rural |  |  | 0.00  1.24 (-2.42, 4.89)  -2.37 (-5.63, 0.90) | 0.04 |
| Two or more hospital admission in past year prior to primary admission |  |  | -3.31 (-5.43, -1.19) | 0.002 |
| MCHC (per 10 g/dL) |  |  | 3.82 (1.24, 6.40) | 0.004 |

Footnote: Coefficients from linear regression to predict hemoglobin at 8 hours and 180 days, adjusted for baseline hemoglobin. N=3,383 for 8 hours and N=2,862 for 180 days.

**Supplementary Table 7.** Multivariable model for second transfusion.

| **Predictor** | **Odds ratio** | **p value** |
| --- | --- | --- |
| Age at admission (per year older) | 1.16 (1.10, 1.21) | <0.001 |
| Height for age z-score at admission (per unit higher) | 1.16 (1.07, 1.27) | <0.001 |
| MUAC for age z-score (per unit higher) | 0.94 (0.83, 1.05) | 0.28 |
| Sickle cell genotype- AA  AS  SS, unknown at discharge  SS, known at discharge | 1.00  0.60 (0.29, 1.24)  0.43 (0.29, 0.63)  0.99 (0.70, 1.41) | <0.001 |
| Blood group- A  B  AB  O | 1.00  1.18 (0.87, 1.60)  0.76 (0.44, 1.31)  0.90 (0.68, 1.19) | 0.19 |
| Received blood transfusion ever, prior to this illness | 1.67 (1.30, 2.14) | <0.001 |
| Type of homestead- Urban  Semi urban  Rural | 1.00  1.91 (1.01, 3.59)  1.86 (1.05, 3.28) | 0.09 |
| Site - Mbale  Mulago  Soroti | 1.00  0.69 (0.48, 1.00)  0.65 (0.48, 0.88) | 0.012 |
| Hemoglobinuria in this illness | 1.90 (1.43, 2.54) | <0.001 |
| Respiratory distress | 1.52 (1.11, 2.07) | 0.009 |
| Impaired consciousness | 1.49 (1.08, 2.04) | 0.01 |
| Oxygen saturation | 0.97 (0.94, 1.00) | 0.03 |
| Splenomegaly- Not palpable  Enlarged  Gross | 1.00  1.06 (0.82, 1.37)  2.04 (1.41, 2.96) | <0.001 |
| Jaundice | 1.30 (1.01, 1.68) | 0.05 |
| Hemoglobin at screening (per g/L) | 0.94 (0.93, 0.95) | <0.001 |
| MCH at admission (per 10 picogram) | 1.45 (1.08, 1.93) | 0.01 |
| Age of blood pack (per day) | 1.03 (1.02, 1.05) | <0.001 |
| Blood pack type - settled (vs whole) | 2.62 (1.97, 3.49) | <0.001 |
| Blood pack hemoglobin (per g/L) | 0.88 (0.85, 0.92) | <0.001 |
| Randomization - 30mls/kg  20mls/kg  Control | 0.34 (0.22, 0.53)  0.68 (0.44, 1.04)  1.00 | <0.001 |

Odds ratios from logistic regression with binary endpoint defined as having a second transfusion. Children who were not transfused were excluded. N=2,984

**Supplementary Table 8.** Univariable summary of outcomes by SCA.

| **Outcome** | **AA N (%) or mean (SD)** | **AS N (%) or mean (SD)** | **AS vs AA p-value** | **Unknown-SCA N (%) or mean (SD)** | **Unknown-SCA vs AA p-value** | **Known-SCA N (%) or mean (SD)** | **Known-SCA vs AA p-value** |
| --- | --- | --- | --- | --- | --- | --- | --- |
| Mortality - 28 days | 85 (4%) | 9 (7%) | 0.053 | 13 (2%) | 0.075 | 11 (3%) | 0.32 |
| Mortality - 180 days | 221 (10%) | 21 (17%) | 0.012 | 35 (6%) | 0.003 | 18 (4%) | <0.001 |
| Readmission - all cause | 411 (18%) | 24 (19%) | 0.63 | 118 (19%) | 0.34 | 72 (17%) | 0.68 |
| Readmission - anemia | 280 (12%) | 18 (15%) | 0.40 | 79 (13%) | 0.53 | 41 (10%) | 0.14 |
| Readmission - malaria | 182 (8%) | 8 (6%) | <0.001 | 24 (4%) | <0.001 | 15 (3%) | <0.001 |
| Time to discharge (days) excluding in-hospital deaths | 3.8 (3.0) | 4.3 (4.2) | 0.79 | 4.0 (4.3) | 0.47 | 3.9 (4.0) | 0.58 |
| Change in Hb - 8 hours | 25 (19) | 24 (20) | 0.78 | 25 (21) | 0.97 | 29 (14) | <0.001 |
| Change in Hb - 180 days | 65 (26) | 58 (29) | <0.001 | 27 (21) | <0.001 | 28 (21) | <0.001 |
| Second transfusion | 362 (17%) | 13 (13%) | <0.001 | 40 (8%) | <0.001 | 99 (23%) | 0.009 |

Descriptive summary of outcomes by SCA category. P-values from exact tests for binary, and Kruskal Wallis tests for continuous outcomes.

**Supplementary Table 9.** Multivariable model for predictors of unknown-SCA (full).

| **Predictor** | **Univariable odds ratio (95% CI)** | **Multivariable odds ratio (95% CI)** | **p value** |
| --- | --- | --- | --- |
| Age (months): ln(age) | 0.62 (0.56, 0.69) | 0.48 (0.37, 0.61) | <0.001 |
| Sex (male vs female) | 0.84 (0.70, 1.01) | 0.70 (0.51, 0.97) | 0.03 |
| MUAC for age z-score at admission (per unit higher) | 0.79 (0.72, 0.86) | 0.82 (0.71, 0.95) | 0.008 |
| HIV positive | 0.32 (0.13, 0.80) | 0.19 (0.04, 0.89) | 0.04 |
| Blood group- A  B  AB  O | 1.00  0.74 (0.56, 0.99)  0.93 (0.59, 1.48)  1.32 (1.05, 1.66) | 1.00  0.66 (0.41, 1.05)  1.18 (0.57, 2.43)  1.31 (0.89, 1.92) | 0.01 |
| Two or more hospital admission in past year prior to primary admission | 0.81 (0.65, 1.02) | 0.77 (0.50, 1.18) | 0.23 |
| Received blood transfusion ever, prior to this illness | 1.30 (1.07, 1.57) | 1.81 (1.25, 2.64) | 0.002 |
| Number of siblings | 0.89 (0.85, 0.92) | 0.90 (0.83, 0.97) | 0.006 |
| Sibling with SCA | 7.06 (4.66, 10.69) | 5.25 (2.68, 10.28) | <0.001 |
| Site - Mbale  Mulago  Soroti | 1.00  2.06 (1.67, 2.54)  1.07 (0.85, 1.34) | 1.00  1.49 (1.02, 2.19)  1.54 (1.00, 2.39) | 0.056 |
| Hemoglobinuria in this illness | 0.29 (0.21, 0.42) | 0.45 (0.27, 0.76) | 0.003 |
| Heart rate: (heart rate/10)^2  (heart_rate/10)^2*ln(heart_rate) | 0.91 (0.88, 0.95)  1.00 (1.00, 1.00) | 0.86 (0.74, 1.00)  1.00 (0.99, 1.01) | 0.01 |
| Temperature | 1.29 (1.17, 1.41) | 1.42 (1.17, 1.71) | <0.001 |
| Systolic blood pressure (per increase of 10) | 1.26 (1.16, 1.37) | 1.27 (1.08, 1.49) | 0.003 |
| History of cough | 0.97 (0.81, 1.16) | 0.68 (0.49, 0.94) | 0.02 |
| Vomiting | 0.50 (0.42, 0.60) | 0.65 (0.47, 0.89) | 0.008 |
| Sunken eyes | 0.60 (0.39, 0.92) | 0.34 (0.13, 0.84) | 0.02 |
| Cold hands | 0.51 (0.28, 0.93) | 1.79 (0.56, 5.71) | 0.32 |
| Splenomegaly- Not palpable  Enlarged  Gross | 1.00  1.05 (0.86, 1.29)  1.73 (1.25, 2.39) | 1.00  1.13 (0.77, 1.65)  1.88 (1.03, 3.42) | 0.12 |
| Jaundice | 0.78 (0.65, 0.95) | 1.35 (0.91, 2.00) | 0.14 |
| Admitted for over 24 hours into another hospital | 0.62 (0.47, 0.82) | 0.55 (0.34, 0.88) | 0.01 |
| Lactate at admission (mmol/L) | 0.92 (0.89, 0.96) | 0.88 (0.82, 0.94) | <0.001 |
| Malaria (rapid diagnostic test or blood slide) | 0.13 (0.10, 0.15) | 0.31 (0.22, 0.44) | <0.001 |
| White blood cells: (wbc/10)  (WBC/10)*ln(WBC/10) | 13.59 (9.41, 19.63)  0.40 (0.34, 0.47) | 0.26 (0.16, 0.41)  712.19 (133.27, 3805.85) | <0.001 |
| MCV at admission (fL) | 1.03 (1.02, 1.03) | 1.04 (1.02, 1.05) | <0.001 |
| Platelets (x 10^9^/L) at admission | 1.01 (1.00, 1.01) | 0.98 (0.97, 0.98) | <0.001 |
| PfHRP2: (PfHRP2/100)  sqrt(PfHRP2/100) | 1.10 (1.07, 1.12)  0.26 (0.22, 0.32) | 1.07 (1.04, 1.09)  0.42 (0.32, 0.54) | <0.001 |

Table presents the model selected for predicting unknown-SCA prior to removing weakly predictive or rarely collected items. Age, heart rate, white blood cells, and PfHRP2 were found to have non-linear effects.
